# Association between social media use and the acceptance of COVID-19 vaccination among the general population in Saudi Arabia – a cross-sectional study

**DOI:** 10.1186/s12889-022-12757-1

**Published:** 2022-02-21

**Authors:** Sahar S. Othman, Abeer Alsuwaidi, Rafal Aseel, Reema Alotaibi, Reem Bablgoom, Ghadeer alsulami, Razan Alharbi, Ranya Ghamri

**Affiliations:** 1 Department of Family Medicine, Faculty of Medicine, King Abdulaziz University, Jeddah, Saudi Arabia; 2 Faculty of Medicine, King Abdulaziz University, Jeddah, Saudi Arabia

**Keywords:** Vaccine hesitancy, Vaccine acceptance, COVID-19, Social media

## Abstract

**Background:**

The Coronavirus Disease of 2019 (COVID-19) pandemic is a worldwide global public health threat. Although acceptance of COVID-19 vaccination will be a critical step in combating the pandemic, achieving high uptake will be difficult, and potentially made more difficult by social media misinformation. This study aimed to examine the association between social media use and acceptance of receiving COVID-19 vaccine among the general population in Saudi Arabia.

**Methodology:**

A cross-sectional study was conducted from June 17 to June 19, 2021 among 504 participants of the general population in Saudi Arabia. The data were collected using a three-part online questionnaire (sociodemographic characteristics, medical and vaccination history, pattern of social media use).

**Results:**

Among 504 participants who completed the survey, 477 participants were acceptant of the vaccine and 27 were non-accepting. A total of 335 individuals had already received the vaccine, 142 were willing to receive the vaccine and 27 were unwilling. One hundred and thirty participants denied using social media for COVID-19 news. Four factors were found to be significant in influencing vaccine acceptance in univariate analysis: having a chronic condition (odds ratio (OR) = 0.367, *P* = 0.019), believing that infertility is a side effect of the COVID-19 vaccine (OR = 0.298, *P* = 0.009), being concerned about a serious side effect from the vaccine (somewhat concerned: OR = 0.294, *P* = 0.022, very concerned: OR = 0.017, *P* < 0.0001), and basing the decision to be vaccinated on social media information (OR = 0.260, *P *= 0.006). Two of these factors fell away on multivariate analysis: basing the decision on social media information (OR = 0.356, *P* = 0.071), and a belief that vaccination causes infertility (OR = 0.0333, *P *= 0.054), suggesting that the associations are dependent on other factors.

**Conclusion:**

‏In conclusion, there was no significant independent relationship between social media usage and people's willingness to receive a COVID-19 vaccination. Further studies to explore the association between social media use and vaccine decisions are required to generalize this observation to the Saudi population.

## Background

The coronavirus disease of 2019 (COVID-19) pandemic is a global public health threat. This unexpected event has caused upheaval all around the world [1]. Governments from all countries have had to act fast to prevent further spread of the virus and thus have implemented preventive measures including restrictions to travel, school closures, social distancing and the mandatory wearing of face masks [2, 3]. It is well recognized, however, that such precautions are insufficient to contain the extension of this viral wave [1]. As a result, vaccine makers have competed to produce vaccines that may potentially curb the spread of COVID-19 and possibly eradicate it [4, 5].

In many countries, including the Kingdom of Saudi Arabia, COVID-19 vaccines are now available [1]. Nevertheless, many people have concerns about receiving vaccines released for emergency use [6].

Attitudes towards COVID-19 vaccinations and their influencing factors have been explored in several studies [7–9]. In China, Fu et al. [8] conducted a study that compared the attitudes of the general public and healthcare workers towards COVID-19 vaccination and found that the latter had a higher level of acceptance [8]. Another study conducted by Thunström et al. [8] in the United States found that only 20% of people would refuse COVID-19 vaccination [8]. In Pakistan, Shah et al. [9] concluded that vaccine hesitancy and rejection were mostly due to religious objection to immunization, security concerns and lack of confidence in the government [9]. More recently, in October 2020, Adebisi et al. [10] studied acceptance of COVID-19 vaccination among social media users in Nigeria. The authors concluded that that most of the respondents were willing to receive COVID-19 vaccination when it became available. The main reasons for not accepting vaccination were the lack of reliability of clinical trials and the belief that the immune system is sufficient to resist the virus [10]. In the United States, COVID-19 vaccine hesitancy has been associated with younger age (< 60 years old), lower levels of education, lower household income, rural residence and lack of health insurance [11–13].

On a local level, a few studies have examined vaccine acceptance and its determinants in Saudi Arabia [14–16]. Findings have shown high hesitancy and low acceptance rates among the Saudi population [15, 16], a potential threat to public health. In the earliest of these studies, Al-Mohaithef et al. [14] concluded that attitudes towards vaccination are context specific and differ according to sociodemographics, culture and geography.

Among the factors previously found to influence the acceptance of vaccination is exposure to vaccine-related misinformation [17], which is largely exchanged in social media [18–20]. Social media networks are web-based applications that allow users to rapidly communicate ideas, thoughts and information with a large audience [21–23]. Social media users present their content as a mixture of evidence, facts and personal opinions that have not been scientifically validated [23, 24]. Furthermore, users can anonymously express their personal opinions [24, 25]. As a result, vaccine-related misinformation is often disseminated on social media [18–20]. Research has shown that exposure to such content has a negative impact on public opinion, leading to vaccine reluctance and hesitancy [26, 27], and this effect has been demonstrated with COVID-19 vaccine acceptance [17]. Loomba et al. [17] examined the effect of exposure to COVID-19 vaccine misinformation on vaccine intent in the United Kingdom and the United States. The authors concluded that misinformation lowers vaccination intent, whereas accurate information does not [17]. In the Middle East, Sallam et al. [28] conducted a study on vaccine apprehension and conspiracy ideas among Jordanians and Kuwaitis, with a small number of participants from other Arab nations, including Saudi Arabia. The authors found that using social media as a primary source of information about COVID-19 vaccination was linked to vaccine apprehension [28]. This finding requires attention and further exploration to help policy makers and guide intervention programs.

To our knowledge, no research has been published that has studied the influence of social media on the acceptance of COVID-19 vaccination among the Saudi population. This study aims to examine the association between social media use and the acceptance of COVID-19 vaccination among the general population in Saudi Arabia. We hypothesize that social media use is associated with vaccine refusal.

## Materials and methods

### Study objectives

The study objectives were:

1. To estimate the rate of willingness to receive COVID-19 vaccination among the general population in Saudi Arabia as of June 2021.

2. To explore factors associated with acceptance of COVID-19 vaccination.

3. To examine the association between social media use and acceptance of COVID-19 vaccination among the general population in Saudi Arabia.

### Study design and data collection

This cross-sectional analytic study used data from a survey that was conducted on the acceptability of (willingness to receive) COVID-19 vaccination among the general population in Saudi Arabia from June 17 to June 19, 2021.

The survey was constructed by the investigators after reviewing the relevant literature. Most of the questions were adopted from previous surveys of similar studies: one study on the acceptability of COVID-19 vaccination [1] and a second one that assessed COVID-19-related fake news on social media [29]. Both sources had open permission to reproduce the research material [1, 29]. The questions were then reviewed independently by two consultants (SO and RG). The survey questions were originally in English. AS, RB, GS and RO translated the questions into Arabic, and RA and RH translated them back to English to ensure that the translation was accurate and preserved the meaning of each question. The survey included questions on demographic data, medical and vaccination history, pattern of social media use, willingness to be vaccinated and reasons for refusal (if applicable). The survey was then distributed to the general population online (in both English and Arabic), using Google Forms through various social media platforms. An online approach was used to avoid physical contact during the pandemic.

As the COVID-19 vaccine was approved by the Ministry of Health during the study period for administration to adults [30–32], our target population was adults 18 years or older who live in Saudi Arabia. Exclusion criteria were individuals less than 18 years old, and those who have a contraindication to receiving COVID-19 vaccination (e.g., allergy to vaccine components).

Prior to the beginning of the survey, online informed consent was obtained from the participants. This included clear information about the study objectives and the target population (eligibility to participate). Participants were clearly informed that the answers they provided would be anonymous and confidential. The informed consent provided two options: ‘yes’ for those who agreed to volunteer and participate in the study and ‘no’ for those who did not wish to participate. Only those who consented and selected ‘yes’ were taken to the questionnaire page to complete the survey.

The sample size was calculated by using the single proportion equation in the Raosoft software package. Based on the assumption that the rate of COVID-19 vaccine acceptance is 50%, and a margin of error of 5% at the 95% confidence level, the required sample size was 385. We collected responses from 504 participants. The snowball sampling technique was employed, and the survey was distributed online to avoid physical contact during the pandemic.

### Measures

The primary outcome variable for this study was acceptance of receiving COVID-19 vaccination. Acceptance was measured by response to the following survey question: ‘Are you willing to take the COVID-19 vaccine?’. Acceptance was defined by indicating ‘yes’ for the question, or by selecting the answer ‘I have already taken the vaccine’. Respondents who responded with ‘no’, i.e., they were unwilling to be vaccinated, were further asked to indicate the main reason(s) for their unwillingness to receive vaccination. The options were: fear of side effects, the vaccine has not been not tested long enough, the vaccine is not effective, and other/ personal reasons.

Other information obtained from the survey was collected as categorical data. First, sociodemographic characteristics were obtained, such as age, gender, marital status, the region of residency, monthly income, education level, and whether the respondent was working in the ‘front line’ in terms of potential exposure to COVID-19. Front-line healthcare workers include all those who are the first contact with patients, such as paramedics, emergency department physicians and nurses, Family physicians, and those who work in COVID-19 swab centers, as well as those who look after COVID-19 inpatients and intensive-care units (ICU). The highest level of education refers to the highest degree obtained by the participant (categorized as Master’s or Doctor of Philosophy (PhD) degree, Bachelor, college diploma, high school certificate, and less than high school). Furthermore, participants were asked to indicate whether they have a degree in healthcare. This refers to graduates of the healthcare programs available in Saudi Arabia; physicians, dentists, nurses, paramedics, pharmacists, laboratory technicians, radiology technicians and physiotherapist. Monthly income in Saudi Riyals (SR) was also categorized into four categories: Less than SR 4000, between SR 4000—SR 10,000, between SR 10,000- SR 20,000, and more than SR 20,000.

In addition to sociodemographic data, information on medical and vaccination history was obtained. This included the presence of any chronic conditions (such as diabetes, hypertension, heart conditions, renal failure, and bronchial asthma), which was a binary variable with a ‘yes’ and ‘no’ options. Vaccination history was composed of previous refusal of vaccines and receipt of influenza vaccine in the past.

Other questions collected information about past infection and perceived risk of getting infected with COVID-19. In addition, participants were asked to indicate which side effects they think are expected after receiving COVID-19 vaccine. They were given the option to choose one or more of the following: Infertility, thrombosis, sudden death, genetic alteration or others.

Finally, information on patterns of social media use was collected, including the use of social media for COVID-19-related news and updates and the preferred social media platform.

### Statistical analysis

Statistical analysis was conducted using IBM SPSS version 20.0 software. Descriptive statistics were presented as frequencies and percentages for the whole sample, and for the two groups of our primary outcome – those acceptant of COVID-19 vaccination and those who were not. The two groups were compared using chi-square test.

The association of each predictor with the outcome (‘acceptance’ vs ‘non-acceptance’ of COVID-19 vaccination) was further tested by conducting univariate binomial regression for each variable. The predictor variables that showed a significant association (*p* < 0.05) with the outcome in the univariate analyses and those with a near significant association (p < 0.1) were entered into a multivariable binary logistic regression model. The level *p* < 0.05 was used as the cut-off value for significance.

### Ethical considerations

This study was designed and conducted in compliance with the ethical principles established by the National Committee of Bio and Medical Ethics at King Abdulaziz City for Science and Technology. Ethical approval was obtained from the Biomedical Research Ethics Committee, Faculty of Medicine, King Abdulaziz University, Jeddah, Kingdom of Saudi Arabia on June 14, 2021 (Reference number 334–21).

## Results

As shown in Table 1, the majority of the 504 participants who completed the online survey were Saudis (*n* = 480, 95.2%), females (*n* = 368, 73%) and residents of the western region (*n* = 345, 68.4%). More than half of the participants (*n* = 289, 57.3%) were from the younger age group, i.e., between 18 and 29 years old, and were educated to Bachelor’s degree (*n* = 287, 56.9%). Among the participants, 153 (30.4%) had a degree in health care, and 59 (11.7%) were frontline healthcare workers. The monthly income for most of the participants (*n* = 237, 47%) was < 4000 Saudi Riyals. The rest of the data on medical and vaccination history is shown in Table 1. Regarding social media use for COVID-19 news and updates, 374 participants (74.2%) responded ‘yes’, whereas the rest (*n*=130, 25.8%) denied using social media for this purpose. Of those who used social media, the majority (*n*= 267, 71.4%) reported that they strictly follow official announcements from health organizations (such as the Ministry of Health or World Health Organization [WHO]), whereas the remaining 107 (28.6%) indicated that they follow any new information available on social media, including expert opinions and personal experiences.

**Table 1:** Frequency distribution of participants’ characteristics according to their acceptance of receiving COVID-19 vaccine (*N* = 504)

Variable	Acceptance of COVID-19 vaccine		Total 504 (100%)
	**Yes** (willing to receive, or have already received the vaccine) 477 (94.6%)	**No** (not willing to receive COVID-19 vaccine) 27 (5.4%)	
**Age (years) **18–29	275 (57.7%)	14 (51.9%)	289 (57.3%)
30–39	84 (17.6%)	4 (14.8%)	88 (17.5%)
40–49	68 (14.3%)	5 (18.5%)	73 (14.5%)
50 +	50 (10.4%)	4 (14.8%)	54 (10.7%)
**Gender**			
Female	349 (73.2%)	19 (70.4%)	368 (73%)
Male	128 (26.8%)	8 (29.6%)	136 (27%)
**Nationality**			
Saudi	454 (95.2%)	26 (96.3%)	480 (95.2%)
Non-Saudi	23 (4.8%)	1 (3.7%)	24 (4.8%)
**Marital status**			
Unmarried	240 (50.3%)	11 (40.7%)	251 (49.8%)
Married	216 (45.3%)	15 (55.6%)	231 (45.8%)
Divorced or widowed	21 (4.4%)	1 (3.7%)	22 (4.4%)
**Residency area**			
Southern region	20 (4.2%)	3 (11.1%)	23 (4.6%)
Eastern region	54 (11.3%)	0 (0%)	54 (10.7%)
Northern region	7 (1.5%)	0 (0%)	7 (1.4%)
Western region	326 (68.3%)	19 (70.4%)	345 (68.4%)
Central region	70 (14.7%)	5 (18.5%)	75 (14.9%)
**Highest level of education**			
**Master's/PhD**	31 (6.5%)	1 (3.7%)	32 (6.3)
Bachelor	274 (57.4%)	13 (48.1%)	287 (56.9%)
College diploma	27 (5.7%)	1 (3.7%)	28 (5.6%)
High School certificate	134 (28.1%)	12 (44.4%)	146 (29%)
Less than a high school diploma	11 (2.3%)	0 (0%)	11 (2.2%)
**Degree in healthcare field**			
No	333 (69.8%)	18 (66.7%)	351 (69.6%)
Yes	144 (30.2%)	9 (33.3%)	153 (30.4%)
**Frontline healthcare worker**			
No	423 (88.7%)	22 (81.5%)	445 (88.3%)
yes	54 (11.3%)	5 (18.5%)	59 (11.7%)
**Monthly income**			
Less than SR 4000	224 (47%)	13 (48.1%)	237 (47%)
Between SR 4000—SR 10,000	140 (29.3%)	7 (26%)	147 (29.2%)
Between SR 10,000- SR 20,000	86 (18%)	6 (22.2%)	92 (18.2%)
More than SR 20,000	27 (5.7%)	1 (3.7%)	28 (5.6%)
**Chronic conditions**			
No	403 (84.5%)	18 (66.7%)	421 (83.5%)
Yes	74 (15.5%)	9 (33.3%)	83-(16.5%)
**Received the seasonal Influenza vaccine in the past**			
No	204 (42.8%)	13 (48.1%)	217 (43.1%)
yes	273 (57.2%)	14 (51.9%)	287 (56.9%)
**Refused vaccination in the past**			
No	405 (84.9%)	25 (92.6%)	430 (85.3%)
Yes	72 (15.1%)	2 (7.4%)	74 (14.7%)
**Infected with COVID-19**			
No	371 (77.8%)	22 (81.5%)	393 (78%)
Yes	106 (22.2%)	5 (18.5%)	111 (22%)
**Family, friends or co-workers infected with COVID-19**			
No	63 (13.2%)	3 (11.1%)	66 (13.1%)
Yes	414 (86.8%)	24 (88.9%)	438 (86.9%)
**Knowing someone passed away due to COVID-19**			
No	190 (39.8%)	12 (44.4%)	202 (40.1%)
yes	287 (60.2%)	15 (55.6%)	302 (59.9%)
**Perceived risk of getting infected with COVID-19**			
Low	202 (42.3%)	10 (37%)	212 (42.1%)
Fair	238 (49.9%)	15 (55.6%)	253 (50.2%)
High	37 (7.8%)	2 (7.4%)	39 (7.7%)
**Social media use for COVID-19 updates and news**			
No	124 (26%)	6 (22.2%)	130 (25.8%)
Yes	353 (74%)	21 (77.8%)	374 (74.2%)
**Preferred media platform for COVID-19 news and updates**			
Twitter			
No	178 (37.3%)	10 (37%)	188 (37.3%)
Yes	299 (62.7%)	17 (63%)	316 (62.7%)
Instagram			
No	395 (82.8%)	20 (74.1%)	415 (82.3%)
Yes	82 (17.2%)	7 (25.9%)	89 (17.7%)
WhatsApp			
No	376 (78.8%)	17 (63%)	393 (78%)
Yes	101 (21.2%)	10 (37%)	111 (22%)
Others*			
No	454 (95.2%)	27 (100%)	481 (95.4%)
Yes	23 (4.8%)	0 (0%)	23 (4.6%)
**Side effects of the COVID-19 vaccine**			
Infertility			
No	432 (90.6%)	20 (74.1%)	452 (89.7%)
Yes	45 (9.4%)	7 (25.9%)	52 (10.3%)
Thrombosis			
No	257 (53.9%)	13 (48.1%)	270 (53.6%)
Yes	220 (46.1%)	14 (51.9%)	234 (46.4%)
Sudden death			
No	397 (83.2%)	19 (70.4%)	416 (82.5%)
Yes	80 (16.8%)	8 (29.6%)	88 (17.5%)
Genetic changes			
No	407 (85.3%)	21 (77.8%)	428 (84.9%)
Yes	70 (14.7%)	6 (22.2%)	76 (15.1%)
Others^b^			
No	256 (53.7%)	19 (70.4%)	275 (54.6%)
Yes	221 (46.3%)	8 (29.6%)	229 (45.4%)
**Concerned about having a serious side effect from COVID-19 vaccine**			
Not concerned at all	266 (%55.8)	5 (18.5%)	271 (53.8%)
somewhat concerned	203 (42.5%)	13 (48.1%)	216 (42.8%)
very concerned	8 (1.7%)	9 (33.3%)	17 (3.4%)
**Decision to vaccinate is based on information from social media**			
No	231 (48.4%)	7 (25.9%)	238 (47.2%)
To some extent	143 (30%)	8 (29.7%)	151 (30%)
Yes	103 (21.6%)	12 (44.4%)	115 (22.8%)

^a^Others: Other social media platforms including Facebook, Snapchat, TikTok, Youtube and Telegram

^b^Others: Other expected side effects of COVID-19 vaccine; including arm pain, fever, headache, malaise and menstrual irregularities

**Fig. 1:**
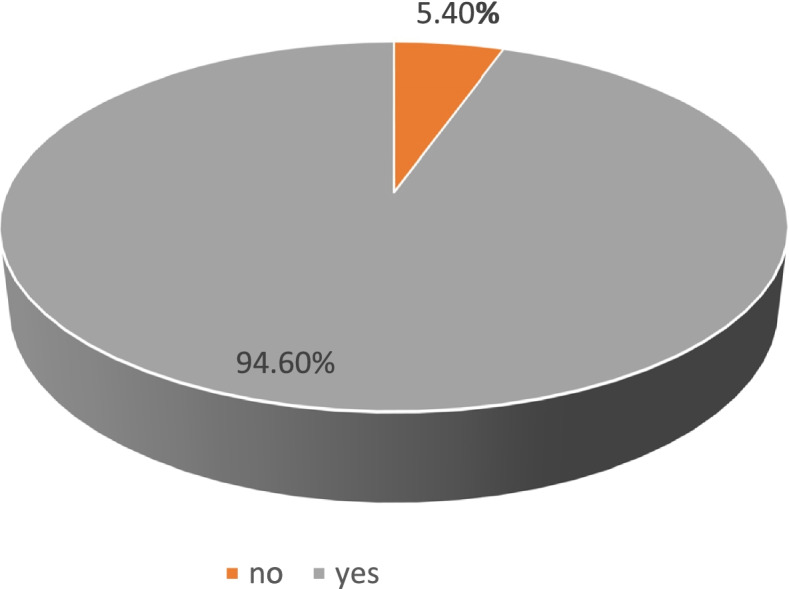
HYPERLINK "sps:id::fig1||locator::gr1||MediaObject::0" Acceptance of COVID-19 vaccine; 94.6% of the participants are acceptant (have already received or are willing to receive COVID-19 vaccine), and 5.4% indicated that they are unwilling to receive COVID-19 vaccine

Among the 504 individuals who completed the survey during the study period, 335 (66.5%) had already received COVID-19 vaccination, 142 (28.2%) indicated that they are willing to receive the vaccine, and 27 (5.4%) indicated that they are not willing to receive the vaccine. In terms of the study’s primary outcome of acceptance of COVID-19 vaccination, 477 participants (94.6%) were acceptant of the vaccine, whereas only 27 (5.4%) of our sample were non-acceptant (Fig. 1). The demographic characteristics, as well as the medical and vaccination information, are presented for both outcome groups in Table 1.

**Fig. 2:**
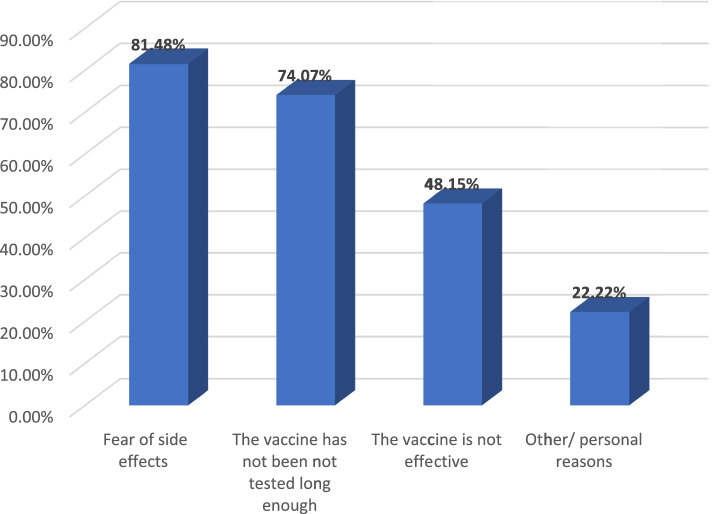
Reasons for refusal of COVID-19 vaccine; among the 27 participants who are unwilling to receive COVID-19 vaccine, the most common reason of refusal was fear of side effects (indicated by 81.48% of participants), followed by the lack of long-term safety data (74.07%)

The 27 participants who responded as not willing to receive the COVID-19 vaccine were further asked about the reasons for refusal. The most common reason was fear of side effects (*n* = 22, 81.5%), followed lack of long-term safety data (*n* = 20, 74.1%). Fig. 2 shows the reasons for COVID-19 vaccine refusal.

To determine predictors that are associated with the study outcome of acceptability of COVID-19 vaccine, each of the variables was first tested independently using univariant binary regression. Of all the study variables, only four were significant: having chronic conditions (OR = 0.367, *P* = 0.019), believing that infertility is a COVID-19 vaccine side effect (OR = 0.298, *P* = 0.009), concern about a serious side effect from the vaccine (somewhat concerned: OR = 0.294, *P* = 0.022, very concerned: OR = 0.017, *P* < 0.0001) and decision to be vaccinated based on social media (OR = 0.260, *P* = 0.006). The results of the univariant binary regression are shown in Table 2. Only the variables that tested as significant (*P* < 0.05) or near significant (*P* < 0.1) are shown. The rest of the variables did not show a significant association with the outcome when tested for single effect. These results were consistent with the chi-square test results. Next, the seven variables that had *P* < 0.1 in the univariant binary regression were entered into a multivariable logistic regression model. As shown in Table 3, two of the significant variables in the univariant regression remained significant in the multivariable model: having chronic conditions (OR= 0.331, *P*=0.036) and concern about a serious side effect from vaccination (very concerned OR=0.019, *P*<0.0001). The other two variables did not show a significant effect when entered into the multivariable model: believing that infertility is a COVID-19 vaccine side effect (OR=0.0333, *P*=0.054), and vaccination decision based on social media (OR=0.356, *P*=0.071). The rest of the variables in the model were all non-significant. The model was overall significant (*P* < 0.001, *R2*=0.302).

**Table 2:** Univariate binary regression estimates of factors associated with acceptance of COVID-19 vaccination

Predictor		***p*-value**	**Odds Ratio** **(OR)**	**95% C.I. for OR**	
				**Lower**	**Upper**
Presence of chronic conditions	yes	0.019	0.367	0.159	0.849
Preferred Media platform for Coronavirus News and updates: What’s App	yes	0.058	0.457	0.203	1.028
Side effects of the COVID-19 vaccine: Infertility	yes	0.009	0.298	0.119	0.742
Side effects of the COVID-19 vaccine: Sudden death	yes	0.093	0.479	0.202	1.131
Side effects of the COVID-19 vaccine: others	yes	0.096	2.050	0.880	4.775
Concerned about serious side effect from the vaccine	not concerned at all		Ref		
	somewhat concerned	0.022	0.294	0.103	0.837
	very concerned	0.000	0.017	0.005	0.061
Decision based on information in social media	no		Ref		
	to some extent	0.246	0.542	0.192	1.526
	yes	0.006	0.260	0.100	0.680

**Table 3:** Multivariate logistic regression estimates of factors associated with acceptance of COVID-19 vaccination

Predictors		*P* value	Odds Ratio (OR)	95% C.I. for OR	
				**Lower**	**Upper**
Presence of chronic conditions	yes	0.036	0.331	0.118	0.931
Preferred Media platform for Coronavirus News and updates: What’s App	yes	0.247	0.564	0.214	1.487
Side effects of the COVID-19 vaccine: Infertility	yes	0.054	0.333	0.109	1.018
Side effects of the COVID-19 vaccine: Sudden death	yes	0.940	0.958	0.317	2.896
Side effects of the COVID-19 vaccine: others	yes	0.231	1.812	0.684	4.797
Concerned about serious side effect from the vaccine	not concerned at all		*Ref*		
	somewhat concerned	0.129	0.430	0.145	1.278
	very concerned	0.000	0.019	0.005	0.080
Decision based on information in social media	no		*Ref*		
	to some extent	0.188	0.454	0.140	1.471
	yes	0.071	0.356	0.116	1.092

## Discussion

Social media plays an important role as a source of information, especially during this pandemic, during which users have sought the most up-to-date information; however, information disseminated via social media can be misleading. The goal of this study was to examine for a link between social media use and acceptance of COVID-19 vaccination in Saudi Arabia's general public.

Most of the respondents of our survey were in younger age groups with more than half of the received responses (57.3%) from individuals between 18 and 29 years old. This response can be explained by the fact that most social media users are young. The majority were vaccinated or acceptant of COVID-19 vaccination. The percentage of the vaccinated individuals was high (66.5%) in comparison to the acceptance rates found in a previous study conducted by Qattan et al. [1] in Saudi Arabia. This compares with findings from the US, where vaccine hesitancy rates also appear to have decreased. In an internet survey of approximately 3500 adults conducted by the Centers for Disease Control (CDC) in September and December 2020, the proportion of adults who reported that they were very likely or absolutely certain to receive a COVID-19 vaccine increased from 39 to 49%, and the proportion who were unlikely to receive one decreased from 38 to 32% [11]. In another survey of approximately 7200 adults, rates of vaccine hesitancy decreased from 46% in October 2020 to 35% in March 2021 [12, 33, 34]. Two reasons explain the significant decrease in rates of vaccine hesitancy. Firstly, as more time lapses following vaccine approval, people become more confident of the safety of vaccination. Secondly, vaccination is becoming mandatory in more places in Saudi Arabia, including for travel, for access to religious/worship settings, as well as schools and commercial buildings [35, 36].

Reasons for unwillingness to be vaccinated, shown in Fig. 2, were fear of side effects (81.5%), insufficient length of time of testing (74.0%), a belief that vaccination is ineffective (48.2%) and other/personal reasons (22.2%). This is consistent with the findings of the CDC survey, where the main reasons for reporting non-intent to receive the vaccine were concerns about its safety and side effects, and a lack of trust in the process [11].

When tested for single effect, four variables showed a significant association with vaccine decision – having a chronic condition, believing that infertility is a side effect, concerns about side effects and making a decision based on social media. All those factors showed a negative effect on the outcome, i.e. making the acceptance less likely, and this result was consistent across analyses. However, deciding based on social media and fear of fertility were not significant when tested for combined effect – only concerns about serious side effects and having a chronic condition remained significantly independently associated with vaccine hesitancy.

The association between having a chronic condition and COVID-19 vaccine acceptance was not significant in an earlier study in Saudi Arabia [37]. However, in this study, the association was significant in both analyses (univariate and multivariate). This association may be explained by a heightened fear of an adverse event or complication following vaccination in the presence of other comorbidities. Concerns of serious side effects is a logical reason for vaccine rejection, and this was significant in both analyses and established in previous studies [38]. Fear of infertility was expressed by some participants, but was not independent of other factors.

A previous study that took place in Riyadh [39] found no significant impact of social media exposure on vaccine hesitancy; however, our findings from the single factor analysis suggest there may be some relationship between social media use and vaccine decision. Our hypothesis was rejected in the multivariate analysis, suggesting that this association is dependent on other factors. Although the internet and social media networks can contribute crucial information, they can also misrepresent the facts, causing distrust and misunderstanding. Our results suggest that healthcare organizations and governments need to provide reliable vaccination information via social media platforms, to influence vaccine hesitancy.

Our study has a number of limitations. It was an observational study conducted at one point in time, therefore no causal relationship between the factors and the outcome can be inferred. Second, the majority of respondents were females and from the western area of Saudi Arabia. The limited response from some areas indicates that the sample was not entirely representative. Finally, the introduction of mandatory vaccination in some contexts in Saudi Arabia potentially confounded or masked the relationships of some variables.

## Conclusion

Although the single factor analysis suggests that there may be some relationship between social media use and COVID-19 vaccine decision, this relationship was found to be dependent on other factors, and no independent association was established. Further studies are required to explore the relationship between social media use and vaccine decisions in order to generalize these observations to the Saudi population.

## Data Availability

The datasets used and/or analysed during the current study are available from the corresponding author on reasonable request.

## Abbreviations

COVID-19: The Coronavirus disease of 2019
OR: Odds ratio
ICU: Intensive-care units
PhD: Doctor of Philosophy
SR: Saudi Riyals
WHO: World Health Organization
CDC: Centre for Disease Control
CI: Confidence interval
